# The Prognostic Value of a Nomogram Model Based on Tumor Immune Markers and Clinical Factors for Adult Primary Glioma

**DOI:** 10.3390/cancers17183043

**Published:** 2025-09-18

**Authors:** Junpeng Wen, Ziling Zhang, Yan Zhao, Yingzi Liu, Jiangwei Yuan, Yuxiang Wang, Juan Li

**Affiliations:** 1 Department of Radiation Oncology, The Fourth Hospital of Hebei Medical University, 12 Jiankang Road, Shijiazhuang 050011, China; wjp13731197930@163.com (J.W.); zlzhang1997@163.com (Z.Z.); zhaoyansjz@hebmu.edu.cn (Y.Z.); 46900849@hebmu.edu.cn (Y.W.); 2 Department of Neurosurgery, The Fourth Hospital of Hebei Medical University, 12 Jiankang Road, Shijiazhuang 050011, China; 47300472@hebmu.edu.cn (Y.L.); yuanjiangwei_doc12@163.com (J.Y.)

**Keywords:** tumor immune markers, immunohistochemistry, glioma, nomogram model, prognosis

## Abstract

**Objective:** This study aimed to identify the factors associated with overall survival (OS) in adult patients with primary gliomas, construct a nomogram prediction model, and evaluate its predictive performance. **Methods:** Clinical data were retrospectively collected from adult patients newly diagnosed with gliomas who underwent surgical treatment in the Department of Neurosurgery of the Fourth Hospital of Hebei Medical University, between January 2019 and December 2023. External validation was conducted using data from the China Glioma Genome Atlas (CGGA) database. Data analysis and visualization were performed using SPSS 26.0 and R software (Version 4.4.1). **Results:** A total of 257 adult patients were included in this study. Multivariate Cox regression analysis identified age, Karnofsky Performance Status (KPS) score, tumor diameter, WHO grade, and postoperative radiotherapy and chemotherapy, as well as the expression of ATRX, IDH1, and Ki-67, as independent prognostic factors. These factors were incorporated into a nomogram for predicting 1-year, 2-year, and 3-year survival rates. The model demonstrated excellent discrimination, calibration, and clinical utility in both internal and external validations. **Conclusions:** The nomogram model incorporating clinical factors (age, WHO grade), treatment (radiotherapy, chemotherapy), and tumor markers (ATRX, IDH1, Ki-67) has good predictive efficacy and may serve as a practical and effective alternative to molecular testing for prediction of survival in adult patients with primary glioma.

## Simple Summary

Glioma is the most common primary malignant tumor of the central nervous system. In the contemporary era of molecular diagnostics for central nervous system tumors, genetic profiling has become essential. However, in certain clinical scenarios—such as stereotactic biopsies, brainstem lesions, or cases involving patients in poor clinical condition who cannot tolerate repeated resections—tumor tissue samples may be insufficient in quantity or exhibit low tumor cellularity. To address these challenges, we developed a novel multi-dimensional prognostic nomogram that integrates tumor immune markers and clinical variables. This tool provides a robust and clinically applicable approach for the individualized management of adult patients with primary glioma.

## 1. Introduction

Glioma is the most common primary malignant tumor of the central nervous system (CNS), accounting for approximately 40–50% of all cases, with an increasing annual incidence rate in adults [1]. Characterized by highly heterogeneous and invasive growth, gliomas exhibit significantly different prognoses among patients with different subtypes [2]. In the molecular era of CNS tumors’ diagnosis, genetic testing has become indispensable. However, in certain clinical scenarios, such as patients undergoing stereotactic biopsy, those with brainstem lesions, or individuals in poor general condition who cannot tolerate secondary surgery, tumor specimens may be limited in quantity or contain a low tumor cell content. This often precludes the extraction of sufficient DNA/RNA for genetic testing. In such cases, immunohistochemistry (IHC) is a better choice [3]. Previous studies have shown that mutations in ATRX, IDH1, and TP53 are significant predictors of glioma prognosis [4,5,6]. Additionally, the expression level of Ki-67 intuitively reflects the proliferative activity of tumor cells [7].

Although molecular typing has improved diagnostic accuracy, dynamic prognostic assessment tools that integrate molecular characteristics with clinical parameters remain lacking in clinical practice. In recent years, nomograms have demonstrated unique value in tumor prognosis models, but current prediction models based on radiomics or genomics are mostly limited to single data dimensions and fail to fully integrate the systematic associations between immunophenotypes, molecular characteristics, and clinicopathological parameters [8,9].

To address this gap, the present study proposes a novel, multi-dimensional prognostic nomogram model that integrates tumor immune markers and clinical factors, aiming to offer a more comprehensive and clinically applicable tool for the individualized management of adult patients with primary glioma.

## 2. Materials and Methods

### 2.1. Study Design and Participants

This study retrospectively analyzed the clinical data of patients newly diagnosed with glioma who underwent surgical treatment at the Department of Neurosurgery of the Fourth Hospital of Hebei Medical University from January 2019 to December 2023. Clinical data were collected from medical records, including gender, age, smoking history, history of head trauma, preoperative Karnofsky Performance Status (KPS) score, symptoms, tumor diameter, tumor location and distribution, extent of surgical resection, WHO grade, postoperative treatment regimen, and protein expression status of ATRX, IDH1, etc.

The inclusion criteria were as follows: (1) patients underwent surgical treatment for the first time in the hospital; (2) complete clinical data records were available; (3) informed consent was obtained from all patients and their families; (4) patients aged ≥ 18 years. The exclusion criteria were as follows: (1) patients with other concomitant malignant tumors; (2) patients with insufficient tumor tissue samples for immunohistochemical testing; (3) patients with prior treatment history for glioma; (4) patients with pathological types of ependymoma, subependymoma, or pilocytic astrocytoma; (5) patients aged < 18 years.

To further validate the reliability and generalization ability of the overall survival (OS) prediction model for glioma patients constructed in this study, an external validation cohort of 100 patients with comparable baseline characteristics was randomly selected from the Chinese Glioma Genome Atlas (CGGA) database [10] (http://www.cgga.org.cn accessed on 7 February 2025).

### 2.2. Histopathological Detection Methods

All tumor tissue samples were formalin-fixed and paraffin-embedded (FFPE) following standard protocols. All histopathological diagnoses were jointly confirmed by two experienced pathologists in our hospital through an independent review of slides. The criteria for positivity were as follows: under the premise of normal negative and positive controls, ATRX protein expression was localized in the nucleus, with positive cells > 10% defined as positive; IDH1-R132H(IDH1) protein expression was localized in the cytoplasm, with ≥10% of tumor cells demonstrating cytoplasmic immunoreactivity being defined as positive [11]; Ki-67 and p53 protein expressions were localized in the nucleus. Positive p53 was defined as > 10% of nuclei containing positive p53 protein granules. A percentage of Ki-67-positive cells ≤ 20% was classified as weak positive (+) and >20% as strong positive (+).

### 2.3. WHO Grading Criteria for Glioma

The diagnosis of gliomas is based on the 2016 edition of the *WHO Classification of Central Nervous System Tumors* [12]. WHO grade II gliomas include IDH-mutant diffuse astrocytomas and oligodendrogliomas; WHO grade III gliomas are anaplastic gliomas, including anaplastic astrocytomas and anaplastic oligodendrogliomas; and WHO grade IV gliomas are mostly glioblastomas.

### 2.4. Treatment of Glioma

Surgical strategies were developed by experienced neurosurgeons based on contrast-enhanced cranial magnetic resonance imaging (MRI) images within 1 week. According to residual tumor volume shown on cranial CT/MRI images 24–72 h postoperatively, the extent of surgical resection was classified into subtotal resection (>80% of tumor volume removed) and partial resection (≤80% of tumor volume removed). Postoperative adjuvant treatments included radiotherapy, concurrent temozolomide chemotherapy, and adjuvant chemotherapy.

### 2.5. Follow-Up

All patients were followed up through a combination of outpatient review and telephone follow-up at 3 to 6 months after surgery. The follow-up deadline was 30 December 2024, with a median follow-up duration of 31.17 months. The OS was defined as the time from the surgery date to the date of death or the last follow-up.

### 2.6. Statistical Analysis

Data analysis and visualization were performed using SPSS 26.0 and R language (version 4.4.1). The Chi-square test was used for categorical variables between groups to determine differences. The ‘linkET’ package in R language was used for visualizing correlation heatmaps, and Spearman correlation analysis was applied for correlation analysis. SPSS 26.0 was utilized for Cox univariate and multivariate analyses [13]. The predictive efficacy of the nomogram was evaluated through ROC curve, consistency index (C-index), calibration curve, and decision curve analysis (DCA), followed by internal and external validation.

## 3. Results

### 3.1. Comparison of Characteristics Among Adult Glioma Patients with Different WHO Grades

**Figure 1:**
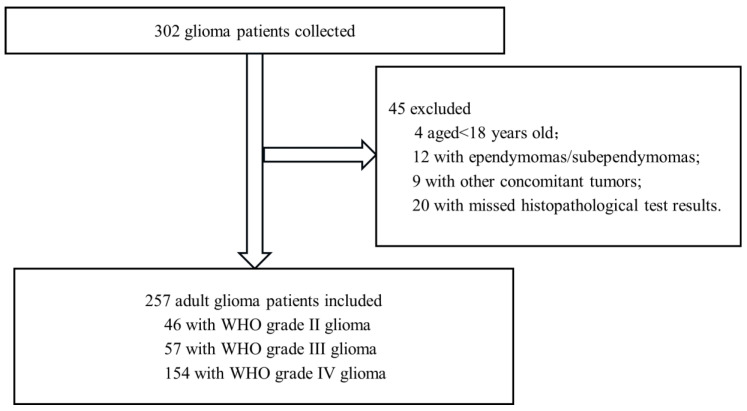
Flowchart of eligible adult patients with primary glioma enrolled in the study.

From January 2019 to December 2023, this study collected clinical data from 302 glioma patients who underwent surgical resection. After excluding 45 cases that did not meet the inclusion criteria (4 patients aged < 18 years old, 8 ependymomas, 4 subependymomas, 9 patients with other concomitant tumors, and 20 patients with missing ATRX, p53, IDH1, or Ki-67 testing results), a total of 257 patients were finally included in the study (Figure 1).

There were significant differences in age at diagnosis, symptoms, KPS score, and tumor location among glioma patients with different WHO grades, while no differences were observed in gender, smoking history, head trauma history, or tumor diameter (Table 1).

### 3.2. Treatment Plan 

The standard treatment for patients with gliomas is maximal surgical resection as recommended by clinical guidelines, followed by adjuvant radiotherapy and/or adjuvant chemotherapy with temozolomide (TMZ) [14,15]. In this study, 154 patients (59.9%) underwent subtotal resection, 80 patients (31.1%) underwent partial resection, and 23 patients (9.0%) received stereotactic biopsy (Table 1). A total of 204 patients received postoperative chemotherapy, and 185 patients received postoperative radiotherapy.

### 3.3. Mutation and Correlation Analysis of Common Immune Markers Across Glioma Grades

**Figure 2:**
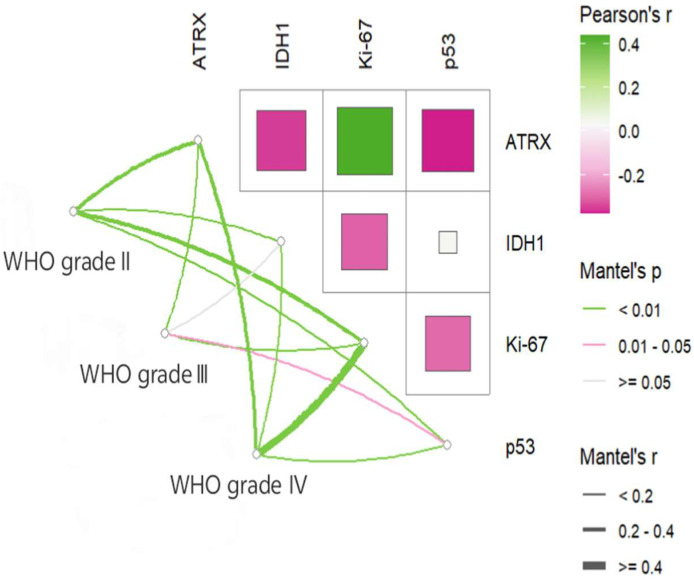
Heatmap of the correlation of common tumor immunomarkers across glioma patients with different WHO grades.

In this study, the mutation positive rates of ATRX, IDH1, and p53 expression levels, as well as the degree of differentiation of Ki-67, varied among glioma patients with different WHO grades, all with statistical significance (Table 1). Correlation analysis was further conducted to explore the associations between the expression levels of these immune markers. As shown in Figure 2, ATRX expression was positively correlated with Ki-67 but negatively correlated with IDH1 and p53. In addition, a significant negative correlation was also observed between Ki-67 and IDH1.

### 3.4. Cox Regression Analysis of Prognostic Factors

**Figure 3:**
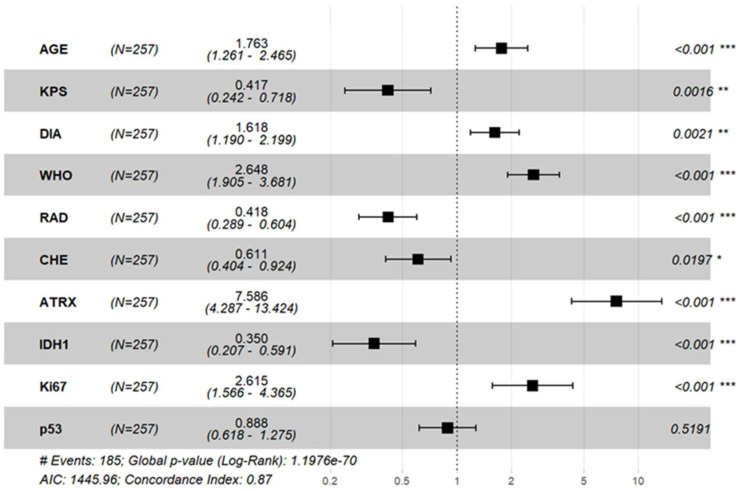
Forest plot of multifactorial Cox regression in 257 patients with glioma. *: control group. Asterisks indicate the level of statistical significance: * *p* < 0.05, ** *p* < 0.01, *** *p* < 0.001.

Univariate Cox regression analysis (Table 2) identified several factors associated with overall survival (OS) in glioma patients, including age at diagnosis, KPS score, WHO grade, postoperative adjuvant radiotherapy, and tumor immune markers (ATRX, IDH1, Ki-67, p53). Variables with statistical significance in the univariate Cox analysis and postoperative chemotherapy were included in the multivariate Cox analysis. As shown in Figure 3, postoperative adjuvant chemotherapy was an influencing factor for glioma prognosis, while P53(−) was no longer a risk factor for poor prognosis in glioma.

### 3.5. Construction and Validation of a Nomogram Prognostic Model

#### 3.5.1. Construction of the Nomogram Prognostic Model

A total of 257 patients were randomly divided into a training set and a validation set at a 6:4 ratio. Meanwhile, 100 patients with balanced baseline characteristics were selected from the CGGA database as an external validation set. There were no significant differences in gender, age, WHO grade, or postoperative chemotherapy, or in the expression of ATRX, IDH1 and Ki-67, among the three groups (*p* > 0.05). However, a significant difference was observed in the proportion of patients receiving postoperative radiotherapy among the three groups (*p* < 0.05), with the external validation set showing a notably higher radiotherapy rate (89%) compared to the training and internal validation sets (Table 3).

**Figure 4:**
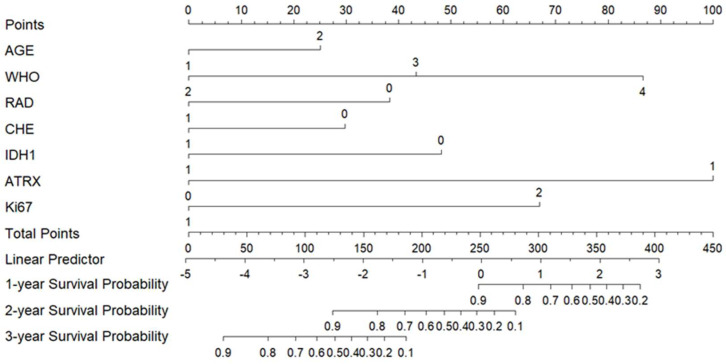
Columnar plot of prognostic OS in patients with gliomas of the brain. Note: AGE: 1 means <60 years old, 2 means ≥ 60 years old; WHO 2, 3, and 4 are WHO grades II, III, and IV respectively; RAD: 1 represents patients who received postoperative radiotherapy, 0 represents patients who did not receive postoperative radiotherapy; CHE: 1 represents patients who received postoperative chemotherapy, and 0 represents patients who did not receive postoperative chemotherapy; IDH1: 0 is negative, 1 is positive; ATRX: 0 is negative, 1 is positive; Ki67: 0 is weak positive, 1 is strong positive.

A prognostic nomogram for predicting 1-year, 2-year, and 3-year survival rates was constructed using variables with *p* < 0.05 identified in multivariate analysis and included in the CGGA external database (Figure 4). The scoring system for each prognostic factor is as follows: age ≥ 60 years: 25.1 points; WHO grade III: 43.3 points; WHO grade IV: 86.6 points; no postoperative radiotherapy: 38.4 points; no postoperative chemotherapy: 29.8 points; IDH1 negative: 48.2 points; ATRX (+): 100 points; Ki-67 (strong+): 67.0 points. The total score was calculated by summing the points of all applicable factors. A higher total score correlates with a worse prognosis (i.e., lower survival probability at 1, 2, and 3 years).

#### 3.5.2. Validation and Evaluation of the Nomogram Prognostic Model

**Figure 5:**
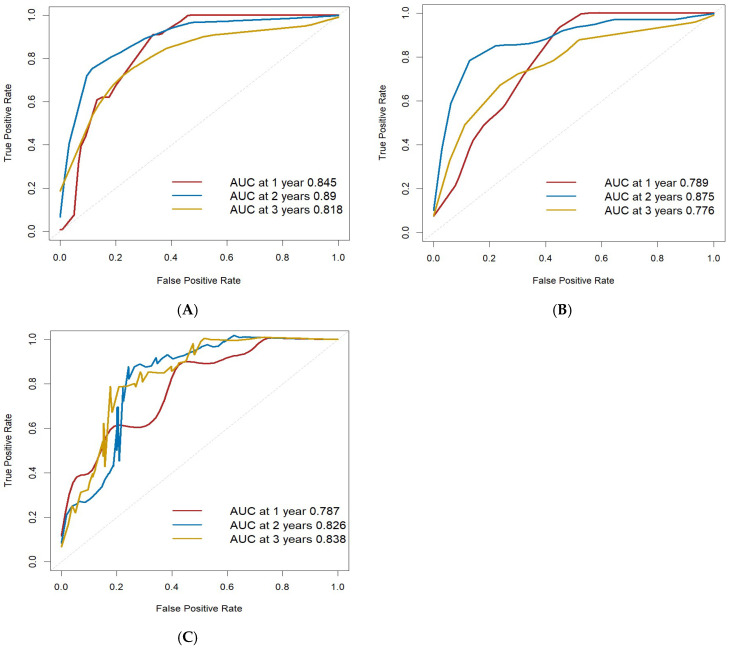
Column line graphs predict ROC curves for 1-, 2-, and 3-year OS in glioma patients. (**A**) ROC curve of training set; (**B**) ROC curve of validation set; (**C**) ROC curve of external validation set.

According to the calculation results of the regression equation, ROC curves were drawn using the data of the training set, validation set, and CGGA external validation set, respectively. The AUC values of 1-year, 2-year, and 3-year OS were all > 0.75 across the three datasets, indicating the good discrimination ability of the model for OS (Figure 5). In addition, the C-index of the training set was 0.861, further supporting the high predictive accuracy of the model.

**Figure 6:**
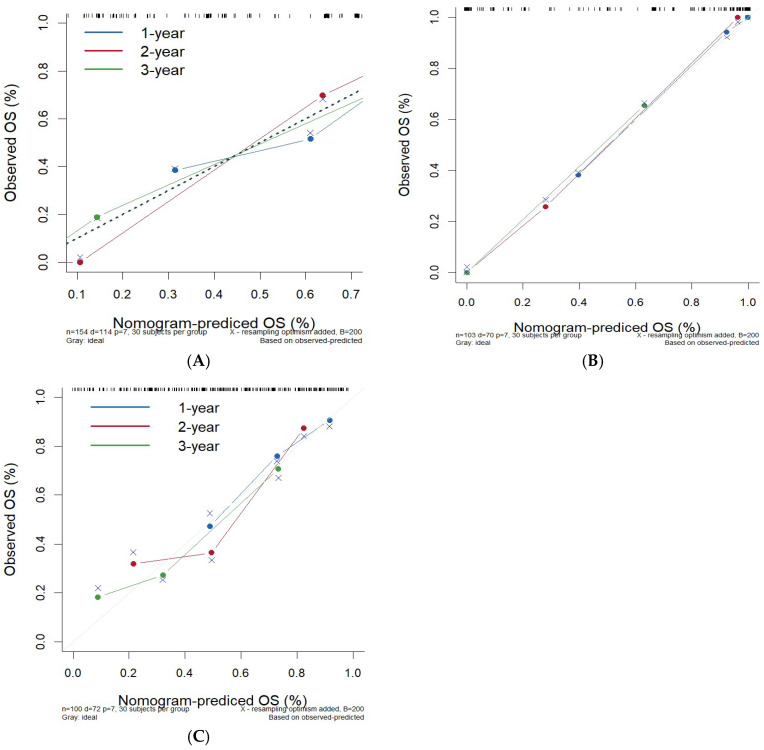
Column line graphs predict calibration curves for 1-, 2-, and 3-year OS in patients with gliomas. (**A**) Calibration curve of training set. (**B**) Calibration curve of validation set. (**C**) Calibration curve of external validation set.

Calibration plots were drawn using the data from the training set, validation set, and CGGA external validation set, respectively. The slopes of the calibration curves in the three cohorts were all close to 1, indicating that the predicted 1-year, 2-year, and 3-year OS rates of the model for glioma patients were basically consistent with the actual values (Figure 6). The calibration ability of the prediction model was evaluated by Hosmer–Lemeshow test. The results showed that χ^2^ = 5.980 and *p* = 0.650 for the model, suggesting that there was no statistical difference between the predicted and observed OS rates, indicating good calibration for the prediction model.

**Figure 7:**
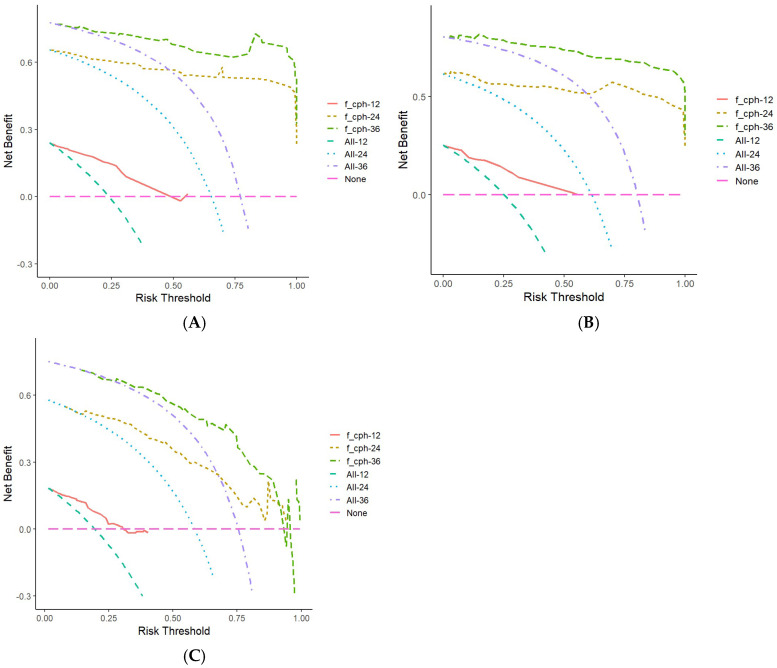
Column line graphs predict decision curves for 1-, 2-, and 3-year OS in glioma patients. (**A**) DCA of training set; (**B**) DCA of validation set; (**C**) DCA of external validation set. Note: The curve labeled ‘f_ cph - 12‘ represents the decision curve analysis for predicting 1-year overall survival (OS) in glioma patients based on a nomogram-derived Cox proportional hazards model, while ‘f_ cph - 24‘ and ‘f_ cph - 36‘ correspond to the decision curves for 2-year and 3-year OS, respectively. The ‘None’ curve serves as a reference for undertaking no intervention.

DCAs were constructed using data from the training set, validation set, and CGGA external validation set (Figure 7). The 1–3-year OS prediction model for glioma patients developed in this study had high net benefit within specific risk threshold ranges. Compared with non-intervention strategies and other comprehensive strategies, the model showed superior clinical utility, suggesting that it can effectively assist in individualized decision-making and provide valuable guidance for clinicians and patients managing glioma.

## 4. Discussion

The fifth edition of the *WHO classification of Central Nervous System Tumors* (2021) proposed an integrated diagnostic model, clarifying the core status of molecular markers in the diagnosis, subtyping, and prognostic assessment of gliomas. By combining histopathological and molecular features, a more accurate diagnostic classification has been established [16]. Synhaeve et al. [17] demonstrated the value of next-generation sequencing (NGS) in identifying glioma subtypes with different prognostic outcomes. Among patients histologically diagnosed as WHO grade II astrocytoma, 16.9% were molecularly diagnosed as WHO grade IV glioblastoma. Although significant progress has been made in the application of molecular characteristics in glioma classification, there still remain some challenges [18]. The survival rate of glioma patients has not been significantly improved in the molecular era, and the high cost of NGS has limited the widespread implementation of large-panel gene testing for Chinese glioma patients in routine post-surgical practice. In this context, IHC detection methods, due to their high efficiency and accessibility, remain an indispensable molecular typing tool in clinical practice [3].

The regulation of the glioma immune microenvironment involves the synergistic action of multi-omics biomarkers. As early as 2009, Yan et al. discovered that patients with IDH-mutated gliomas had significantly better prognoses. IDH mutations are more common in low-grade gliomas and secondary glioblastomas [19]. Multiple studies on IDH inhibitors for the treatment of gliomas have been conducted [20,21], which are expected to change the treatment regimen for IDH-mutated gliomas. In adult primary gliomas, IDH-mutant gliomas are often accompanied by ATRX mutations [22]. Olar et al. [23] showed that patients with co-mutations had higher survival rates. Hu et al. [24] found that ATRX mutations activate the BRD-dependent immunosuppressive transcriptome and immune escape mechanisms in IDH1 R132H-mutated astrocytoma cells. Additionally, Murnyak et al. [25] found that gliomas with IDH1 mutations are often accompanied by TP53 mutations. The TP53 gene plays a key role in processes such as cell cycle regulation, DNA damage repair, and apoptosis [26]. However, the relationship between TP53 gene mutation status and survival outcomes remains inconclusive. Some studies [27,28] have reported that patients with TP53 mutations have better survival rates, while others have not found such a correlation [29]. Xie et al. [6] found that ATRX mutations often coexist with IDH1 and TP53 mutations in low-grade gliomas, raising the possibility of interactions among these genes and ferredoxin reductase (FDXR). Nonetheless, the precise synergistic mechanisms underlying ATRX, IDH1, and TP53 mutations remain poorly understood and warrant further investigation at the molecular level.

Hu et al. [11] demonstrated a negative correlation between IDH1 and Ki-67, a finding corroborated by our analysis of commonly used glioma biomarkers. The expression level of Ki-67 reflects the proliferative activity of tumor cells [7], and tumor cells with high proliferative activity are more likely to lead to tumors’ recurrence and metastasis [30]. Several studies [31,32] have shown that glioma patients with weak positive Ki-67 have a longer OS. Therefore, by detecting the positive expression rate of Ki-67 in tumor tissues, the proliferative status of tumor cells can be evaluated.

Nevertheless, the prognosis of gliomas is not determined by a single immune marker alone. Factors such as patient age, KPS score, tumor size and location, extent of surgical resection, postoperative treatment regimen, and expression of immune markers interact with each other in a complex manner to determine the patient’s prognosis [33,34].

Gong [35] et al. developed a homologous recombination deficiency (HRD) score-based prognostic model for glioma, which identified seven signature genes via machine learning and demonstrated superior predictive performance across multiple cohorts. The study also revealed correlations between HRD scores, genomic instability, and immune infiltration patterns, providing potential insights for immunotherapy and personalized treatment strategies. Huang et al. [36] developed a fusion prognostic model combining radiomics and clinical factors, which demonstrated optimal performance in predicting survival for patients with diffuse glioma. Based on this model, they constructed an accessible ‘Prognosis Calculator for Diffuse Glioma’ to assist clinical decision-making.

In this study, a nomogram model was developed by integrating tumor immune markers (ATRX, IDH1, Ki-67) and clinical characteristics (age, WHO grade, postoperative radiotherapy and chemotherapy). This model enables a comprehensive assessment of the prognosis in adult patients with primary glioma. Compared with traditional evaluation methods, the nomogram model considers the interaction of multiple factors and provides more accurate and individualized prognostic information. ROC curves were drawn using the data from the training set, validation set, and CGGA external validation set, respectively, with all AUC values greater than 0.75, indicating robust discriminatory performance. Calibration curves for the training set, validation set, and CGGA external validation set were plotted, and the slopes of the calibration curves were all close to 1, demonstrating strong concordance between predicted and observed outcomes. Furthermore, the DCA curve confirmed the clinical utility of the nomogram, suggesting its potential value in guiding treatment decisions and facilitating shared decision-making between clinicians and patients.

This study still has some limitations. First, the relatively small sample size may affect the accuracy and generalizability of the nomogram. Although strict inclusion and exclusion criteria were adopted in the model’s construction, potential selection bias cannot be entirely excluded. Second, external validation was limited to the CGGA database. Although the validation results show that the model has a good predictive performance, prospective, multi-center clinical studies are needed to further confirm its robustness. Third, due to data constraints, we were unable to incorporate additional tumor biomarkers (such as MGMT promoter methylation, TERT, etc.). We plan to include these in future studies. Fourth, there was a significant difference in the rate of radiotherapy’s administration between the development and external validation cohorts. This difference represents a potential source of confounding, as radiotherapy is a key determinant of patient outcomes. Unfortunately, due to the lack of detailed data on the specific indications for radiotherapy (e.g., margin status, extracapsular extension) in the external validation cohort, we were unable to adjust for this critical variable. Although our model maintained its performance in the validation set, we cannot fully rule out that the difference in treatment rates may have influenced the results.

Future research could identify additional prognostic biomarkers for adult primary gliomas. While the integration of pathological factors and molecular characteristics remains the optimal reference for diagnosis and treatment at present, the advent of IDH inhibitors has markedly improved survival outcomes in patients with IDH-mutant gliomas, establishing a new standard of care for this subset of tumors [20,21]. Future studies should prioritize validating and refining our prognostic model within this evolving therapeutic context, particularly to assess its performance in predicting treatment response and long-term survival among patients receiving IDH-targeted therapy. Furthermore, investigating the interaction between model-derived risk strata and the efficacy of IDH inhibitors may offer valuable insights for personalized treatment strategies and patient stratification in clinical trials.

## 5. Conclusions

This nomogram model incorporating clinical factors (age, WHO grade), treatment (radiotherapy, chemotherapy), and tumor markers (ATRX, IDH1, Ki-67) has a good predictive efficacy and may serve as a practical and effective alternative to molecular testing for prediction of survival in adult patients with primary glioma.

## Figures and Tables

**Table 1:** Comparison of clinical characteristics among adult patients with different WHO grades of glioma.

	*n*	WHO II	WHO III	WHO IV	χ^2^	*p *Value
Gender						
male	143	23	35	85	1.372	0.503
female	114	23	22	69		
Age (y)						
<60	137	39	38	60	35.130	<0.001
≥60	120	7	19	94		
Smoking history						
yes	86	16	22	48	1.075	0.584
no	171	30	35	106		
History of head trauma						
yes	20	4	3	13	0.651	0.722
no	237	42	54	141		
KPS score						
≥80	237	46	56	135	11.221	0.004
<80	20	0	1	19		
Diameter (cm)						
<5	157	32	34	91	1.699	0.428
≥5	100	14	23	63		
Symptom						
intracranial hypertension	100	15	22	63	29.090	<0.001
neurological dysfunction	100	10	22	68		
epilepsy	39	16	10	13		
mental disorders	8	1	0	7		
no obvious symptoms	10	4	3	3		
Tumor distribution						
left side	123	24	27	72	3.695	0.718
right side	97	15	25	57		
bilateral	21	4	4	13		
middle	16	3	1	12		
Tumor location						
frontal lobe	101	28	24	49	25.461	0.001
temporal lobe	63	4	11	48		
parietooccipital lobe	53	8	8	37		
insular lobe	14	0	6	8		
others	26	6	8	12		
Surgical type						
subtotal resection	154	33	30	91	5.371	0.251
partial resection	80	10	23	47		
stereotactic biopsy	23	3	4	16		
Postoperative chemotherapy						
yes	204	32	43	129	5.058	0.080
no	53	14	14	25		
Postoperative radiotherapy						
yes	185	3	12	57	18.090	<0.001
no	72	43	45	97		
ATRX						
positive	171	10	26	135	83.536	<0.001
negative	86	36	31	19		
IDH1						
positive	62	24	17	21	30.038	<0.001
negative	195	22	40	133		
p53						
positive	106	29	31	46	21.304	<0.001
negative	151	17	26	108		
Ki67						
weakly positive	84	42	32	10	134.111	<0.001
Strong positive	173	4	25	144		

**Table 2:** One-way Cox prognostic analysis of overall survival and progression-free survival in 257 patients with glioma.

	OS		
	HR	95%CI	*p* Value
Gender (male/female)	0.913	0.683–1.220	0.540
Age (<60/≥60)	3.502	2.555–4.800	<0.001
Smoking history (no/yes)	0.799	0.585–1.092	0.159
KPS score(<80/≥80)	0.114	0.069–0.188	<0.001
Diameter (<5 cm/≥5 cm)	1.514	1.129–2.030	0.006
History of head trauma (no/yes)	1.145	0.674–1.944	0.616
WHO grade			
WHO II	1 *		0 *
WHO III	3.477	1.784–6.775	<0.001
WHO IV	14.739	7.995–27.170	<0.001
Postoperative radiotherapy (no/yes)	0.281	0.204–0.388	<0.001
Postoperative chemotherapy (no/yes)	0.739	0.512–1.068	0.107
ATRX (−/+)	11.042	7.083–17.213	<0.001
IDH1 (−/+)	0.221	0.140–0.350	<0.001
p53 (−/+)	0.475	0.349–0.646	<0.001
Ki67 (weak+/strong+)	6.611	4.402–9.929	<0.001

* Control group.

**Table 3:** Comparison of clinical characteristics between training set, validation set, and CGGA external validation set.

	Training Set (*n* = 154)	Validation Set (*n* = 103)	CGGA (*n* = 100)	χ^2^	*p*
Gender					
male	91 (59.1%)	50 (48.5%)	61 (61.0%)	3.898	0.142
female	63 (40.9%)	53 (51.1%)	39 (39.0%)		
Age (year)					
<60	78 (50.6%)	56 (54.4%)	52 (52.0%)	0.343	0.843
≥60	76 (49.4%)	47 (45.6%)	48 (48.0%)		
WHO grade					
WHO II	28 (18.2%)	19 (18.4%)	18 (18.0%)	1.038	0.904
WHO III	31 (20.1%)	26 (25.2%)	22 (22.0%)		
WHO IV	95 (61.7%)	58 (56.3%)	60 (60.0%)		
Postoperative radiotherapy					
yes	112 (72.7%)	75 (72.8%)	89 (89.0%)	10.821	0.004
no	42 (27.3%)	28 (27.2%)	11 (11.0%)		
Postoperative chemotherapy					
yes	121 (78.6%)	77 (74.8%)	69 (69.0%)	2.946	0.229
no	33 (21.4%)	26 (25.2%)	31 (31.0%)		
ATRX					
negative	45 (29.2%)	38 (36.9%)	37 (37.0%)	2.342	0.310
positive	109 (70.8%)	65 (63.1%)	63 (63.0%)		
IDH1					
negative	113 (73.4%)	86 (71.8%)	70 (70.0%)	0.344	0.842
positive	41 (26.6%)	171 (28.2%)	30 (30.0%)		
Ki-67					
weak+	50 (32.5%)	35 (34.0%)	30 (30.0%)	0.376	0.829
strong+	104 (67.5%)	68 (66.0%)	70 (70.0%)		

## Data Availability

The data supporting the findings of this study are publicly available in the Chinese Glioma Genome Atlas (CGGA) at http://www.cgga.org.cn (accessed on 7 February 2025) and can be accessed with the dataset identifiers [mRNAseq_325].
